# Efficiency Improvement of HIT Solar Cells on p-Type Si Wafers

**DOI:** 10.3390/ma6115440

**Published:** 2013-11-22

**Authors:** Chun-You Wei, Chu-Hsuan Lin, Hao-Tse Hsiao, Po-Chuan Yang, Chih-Ming Wang, Yen-Chih Pan

**Affiliations:** 1 Department of Opto-Electronic Engineering, National Dong Hwa University, Shoufeng, Hualien 97401, Taiwan; E-Mails: c5170442@gmail.com (C.-Y.W.); a3747@livemail.tw (H.-T.H.); wangcm@mail.ndhu.edu.tw (C.-M.W.); cypress1228@gmail.com (Y.-C.P.); 2 15F., No. 23-10, Wenhua Rd., Xindian District, New Taipei City 23144, Taiwan; E-Mail: f92943037@ntu.edu.tw

**Keywords:** HIT, solar cells, p-type Si substrate

## Abstract

Single crystal silicon solar cells are still predominant in the market due to the abundance of silicon on earth and their acceptable efficiency. Different solar-cell structures of single crystalline Si have been investigated to boost efficiency; the heterojunction with intrinsic thin layer (HIT) structure is currently the leading technology. The record efficiency values of state-of-the art HIT solar cells have always been based on n-type single-crystalline Si wafers. Improving the efficiency of cells based on p-type single-crystalline Si wafers could provide broader options for the development of HIT solar cells. In this study, we varied the thickness of intrinsic hydrogenated amorphous Si layer to improve the efficiency of HIT solar cells on p-type Si wafers.

## 1. Introduction

Solar cells have been in active development for many years as an alternative energy source. One leading commercialized technology is the high efficiency HIT (heterojunction with intrinsic thin layer) solar cell devised by Sanyo on n-type crystalline silicon (c-Si) wafers [1]. This promising structure has the following advantages: (1) simple structure without the need for complicated fabrication technologies; (2) with the simple structure allows for a low-temperature fabrication process, resulting in longer minority carrier lifetimes, and superior performance; and (3) the inserted intrinsic layer suppresses surface recombination [2,3,4]. Our aim is to study the HIT structure and apply it to p-type c-Si wafers in order to extend possible applications. For both p and n type substrate HIT structures, a discussion of the band diagram is quite important for understanding how carriers move and are collected to optimize cells. Later, we present and explain the p-type Si band structure for our research. Although n-type wafers are usually used in commercial HIT cells, most researchers still concentrate on HIT cells on p-type wafers [5]. In the view of efficiencies, the p-type wafers are not as suitable as n-type wafers for HIT cells. The initial efficiencies of p-type wafers are lower and they continuously decrease for ~3% due to the boron-oxygen complex [6]. However, in the view of cost, the market based on p-type wafers has the potential to grow up. Up to now, the material cost of p-type wafers is still lower than n-type wafers [7]. Hence, if the efficiencies based on p-type wafers can be further improved, the topics focusing on p-type wafers should be continuously considered. In this manuscript, we find the method to increase the efficiencies of p-type wafers. The method cannot be performed on n-type wafers.

In order to consider the optical characteristics of the cells, we used the optical simulation tool, R-SOFT to include the effect of reflection and scattering of incident light. This allowed us to understand how much of the incident light is reflected at different wavelengths. Then, with the modified input light spectrum, the current-voltage characteristics obtained by the electrical simulation tool, Sentaurus TCAD, were similar to results obtained in practical cells. Based on the results, we varied the thickness of the top intrinsic hydrogenated amorphous Si [a-Si:H(i)] layer to improve the efficiency of HIT solar cells on p-type Si wafers. The single crystal Si wafer was first assumed to have a typical thickness of 250 µm in the HIT cell. We also tested single-crystalline Si of 1.5 μm thickness. Such thin single-crystalline Si is possible due to smart-cut technology [8,9]; thus, material consumption can be reduced significantly. The enhancement due to the increase of the a-Si:H(i) layer is far more significant when a thinner single crystal Si wafer is used.

## 2. Device Structure

**Figure 1:**
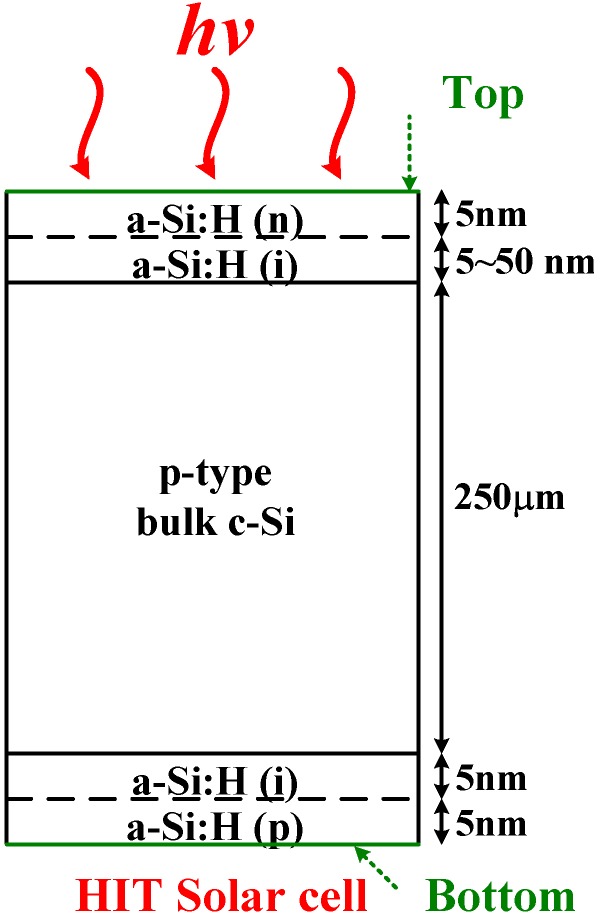
Schematic structure of an HIT solar cell with p-type crystalline Si. The a-Si:H(i) layer can reduce the surface recombination rate.

Figure 1 is a schematic structure of an HIT solar cell, and the middle bulk c-Si is p-type and 250 µm thick. There is 5 nm thick intrinsic amorphous Si (a-Si) at the back of the bulk c-Si. There is also an intrinsic a-Si layer on the front of the bulk c-Si, and its thickness was varied from 5 to 50 nm to study the influence of the top intrinsic layer. The top layer was 5 nm thick n-type a-Si, and the bottom layer was 5 nm thick p-type a-Si. In production, the a-Si on c-Si is usually deposited using plasma-enhanced chemical-vapor-deposition (PECVD) [10]. The bottom a-Si layer with impurities can provide the back surface field [11,12], which is used for lowering the surface recombination phenomenon. The intrinsic a-Si layers play very important roles within the a-Si layers, lowering defects at the interfaces between highly-doped a-Si and c-Si layers, and boosting conversion efficiencies. Without the intrinsic a-Si thin layers, recombination via defects in the highly-doped a-Si would be significant.

**Figure 2:**
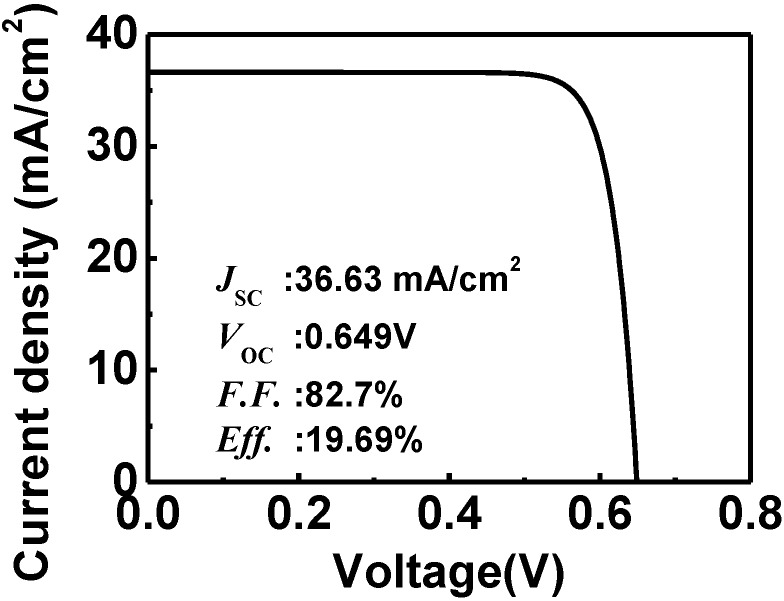
Current density *versus* voltage of the HIT solar cell on the p-type Si wafer. The *V*_oc_ is 0.649 V, and the *J*_sc_ is 36.63 mA/cm^2^.

In practical cells, the top surface would be textured to reduce wastage of input light due to reflection. The textured structure is modeled by pyramids with width and depth of 0.1~1 µm using the optical simulation tool, R-SOFT. After considering the reflection (R) as a function of the wavelength, the AM 1.5 G spectrum is multiplied by the factor (1-R). The grid electrode leads to a shading loss of ~7%, and it is also included in the modified spectrum. With the corrected light spectrum, the current density *versus* voltage (*J*–*V*) curve can be obtained using the electrical simulation tool, Sentaurus TCAD, and this curve is shown in Figure 2. In this example, the top intrinsic a-Si layer is set at the common thickness of 5 nm. This *J*–*V* curve shows that the HIT structure has an efficiency of 19.69%. For comparison, the efficiencies of recent HIT solar cells on the p-type FZ wafer and the p-type CZ wafer are 19.3% and 18.8%, respectively [13]. Our simulation result is similar to results obtained in these practical cells.

## 3. Results and Discussion

**Figure 3:**
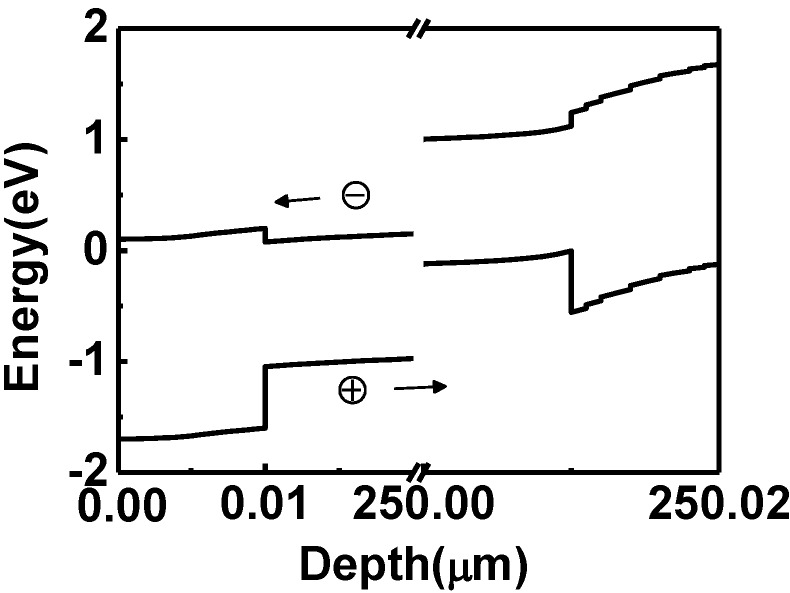
The band structure of an HIT solar cell on a p-type Si wafer with a 5 nm-thick top i-layer.

The band structure of the HIT solar cell on the p-type Si wafer is shown in Figure 3. According to this diagram, the photo-generated electrons will be swept to the top surface by the built-in potential. The conduction band offset at the front a-Si/c-Si interface is small, and almost all electrons can easily overcome this offset. The valence band offset at the back a-Si/c-Si interface is large, so the back a-Si(i) layer should be thin enough for hole tunneling in order to suppress blocking by the large offset. Fortunately, we only need to increase the thickness of the top a-Si(i) layer to enhance the absorption and collection of photo-generated carriers contributed by short-wavelength light. The valence band offset at the front hetero-interface is large, but it does not prevent the photo-generated holes from being collected since generated holes are collected to the back surface. In contrast to our p-type c-Si HIT cell, increase in the thickness of the top a-Si(i) layer of the traditional Sanyo’s p/i a-Si–n c-Si–i/n a-Si HIT cell will prevent the collection of photo-generated holes. Hence, the investigation of the benefit from increasing the thickness of a-Si(i) is focused on the HIT cell on p-type wafers.

**Figure 4:**
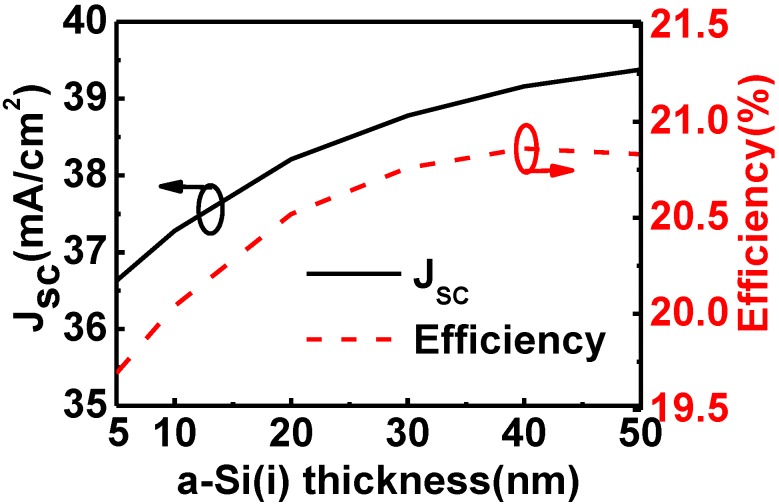
Short-circuit current density and efficiency of HIT solar cells as a function of the thickness of the top a-Si:H(i) layer when the bulk c-Si was 250 μm thick.

We investigated the short-circuit current densities (*J*_SC_) and efficiencies of the HIT solar cells on p-type wafers as a function of the thickness of the top a-Si:H(i) layer when the bulk crystalline Si was 250 µm thick (Figure 4). The results indicated that as the thickness increased, the *J*_SC_ also increased due to increased absorption of short-wavelength light in the top a-Si:H(i) layer. The *J*_SC_ increased from 36.63 mA/cm^2 ^in the 5 nm thick a-Si:H(i) case to 39.16 mA/cm^2^ in the 40 nm-thick a-Si:H(i) case. The corresponding efficiency increase was from 19.69% to 20.86%. Beyond 40 nm, greater absorption of short-wavelength light makes the *J*_SC_ increase to 39.38 mA/cm^2^ when the a-Si(i) is 50 nm thick. However the efficiency started to decrease as thickness increased as a result of lower mobility in the a-Si degrading the fill factor. The resulting efficiency with a 50 nm thick a-Si(i) layer was 20.83%.

**Figure 5:**
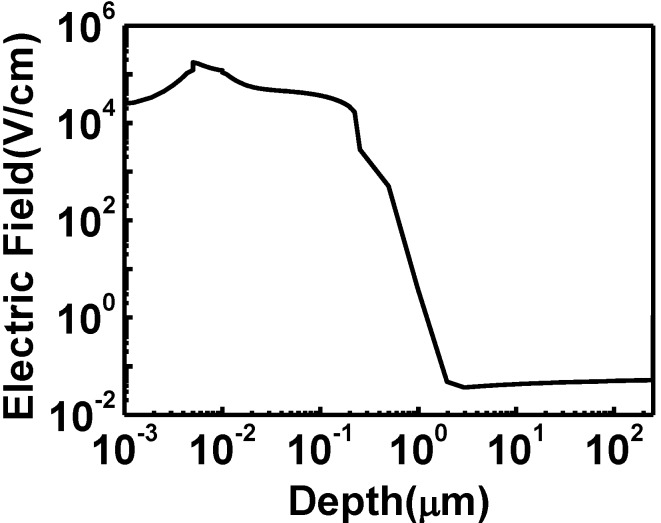
The electric field distribution within the HIT structure. The electric field is largest within the top intrinsic a-Si:H layer.

Figure 5 shows the electric field distribution within the HIT structure with 250 µm thick bulk c-Si and a 5 nm thick top a-Si(i). From this figure, we found that the highest electric field region was mostly within the top a-Si:H(i) layer. The high electric field could aid the separation of photo-generated carriers without recombination.

For comparison, simulation results of the conjugate cell structure with the n-type wafer can be found in [14]. In that p/i a-Si–n c-Si–i/n a-Si cell, the thicker top a-Si (i) layer would result in a smaller *J*_SC_ due to the prevention of carrier collection at the top a-Si(i)/c-Si interface [14]. On the other hand, the increase on the thickness of the top a-Si(i) layer of the n/i a-Si–p c-Si–i/p a-Si cell in this manuscript would not prevent the carrier collection, since the carrier flow of this case is in the different direction. In practical cases, the transparent conductive oxide (such as ITO) is usually used on the top of a-Si layers, and this degenerated oxide should be considered to obtain a more precise simulation [15]. With ITO considered, as described in [15], tunneling is only significant at the bottom side for the cell with the p-type wafer. This conclusion is consistent with our results. Hence, the increase of the top a-Si (i) layer for the p-type crystalline Si cell would not impede carrier collection but has the probability to improve the efficiency.

**Figure 6:**
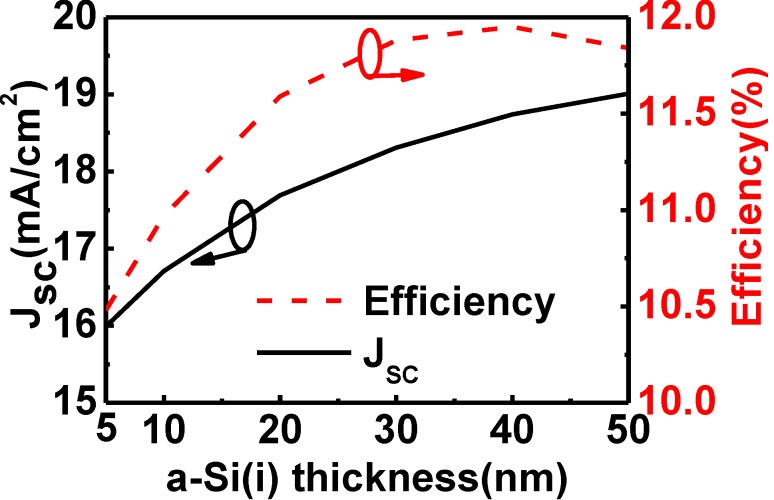
Short-circuit current density and efficiency of HIT solar cells as a function of the thickness of the top a-Si:H(i) layer when the bulk c-Si was 1.5 µm thick.

We also investigated the influence of the thickness of a-Si:H(i) when the bulk crystalline Si was only 1.5 µm thick. In Figure 6, we see that the thicker the i-layer, the larger is the *J*_SC_ as a result of greater absorption of short-wavelength light in the top a-Si:H(i) layer. The *J*_SC_ increased from 15.99 to 18.74 mA/cm^2 ^when the thickness of the a-Si:H(i) was increased from 5 to 40 nm; the efficiencies increased from 10.48% to 11.95%. When the 50 nm-thick top a-Si(i) layer was used the *J*_sc_ increased to 19.01 mA/cm^2^, and the efficiency decreased to 11.84%. The trend of both curves is similar to Figure 4. The ratio of the performance enhancement due to the increase of the top a-Si(i) was greater in the cell with 1.5 µm thick c-Si than with 250 µm thick c-Si. Hence, if the HIT structure is applied to the p-type thin-film c-Si due to the lower material cost and better temperature coefficient [16], its top a-Si(i) layer should be thickened.

## 4. Summary

In summary, we have characterized the relationship between the thickness of the active layers and the performance of the HIT solar cells based on p-type single crystalline Si wafers. The high electric field in the top amorphous layer can aid in the collection of carriers generated by short-wavelength light. We have shown that an increase in the thickness of the top intrinsic layer has the potential to significantly improve cell performance.
